# Un angiobehçet: entre le marteau et l'enclume!

**DOI:** 10.11604/pamj.2015.21.223.7404

**Published:** 2015-07-30

**Authors:** Imène Boukhris, Samira Azzabi

**Affiliations:** 1 Service de Médecine Interne B, Hôpital Charles Nicolle, Tunis, Tunisie

**Keywords:** Behçet, thrombose veineuse cérébrale, hémorragie digestive, Behçet, cerebral venous sinus thrombosis, gastrointestinal bleeding

## Image en medicine

L'atteinte vasculaire est fréquente au cours de la Maladie de Behçet (MB). Dans 1/3 des cas, il peut s'agir d'une thrombose veineuse des gros troncs. L'atteinte artérielle peut survenir dans 4 à 17% des cas. Le traitement de ces atteintes graves d'angiobehçet repose sur les corticoïdes et les immunosuppresseurs. L'intérêt des anticoagulants reste controversé. Nous rapportons un cas de thromboses artérielles et veineuses insolites et graves entrant dans le cadre d'une MB, chez un patient à haut risque hémorragique. Il s'agissait d'un patient âgé de 43 ans, présentant une MB avec une hypertension intracrânienne, une atrophie optique bilatérale et une cécité. Le patient présentait un angiobehcet fait de lésions vasculaires thrombotiques artérielles et veineuses avec de multiples localisations graves insolites et récidivantes: une thrombophlébite cérébrale, un antécédent d'AVC ischémique hémisphérique droit en rapport avec une occlusion de la carotide interne droite, une thrombophlébite du sinus latéral gauche, une thrombose veineuse jugulaire interne droite, un infarctus mésentérique, une thrombose portale avec cavernome porte compliqué d'une hypertension portale et d'hémorragie haute par rupture de varices œsophagiennes. Un traitement anticoagulant a été discuté mais récusé devant: le haut risque hémorragique des varices œsophagiennes persistantes après une ligature et l'ancienneté des lésions thrombotiques. Un traitement de fond à base d'Imurel^®^ était prescrit. Devant l'ancienneté des lésions qui ne paraissent pas actives à l'angio-IRM cérébrale de contrôle, une corticothérapie n'a pas été indiquée. Les atteintes présentées par ce patient, loin d’être rares, sont particulièrement graves, assombrissant nettement le pronostic de la maladie.

**Figure 1:**
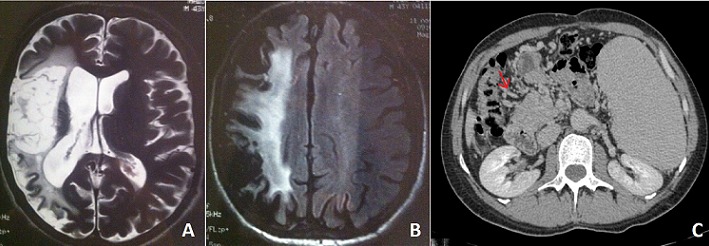
(A) IRM cérébrale en séquence axiale T2 et (B) en séquence axiale T2 flair: montrant une cavité porencéphalique hémisphérique droite séquellaire avec anomalie de signal corticosous corticale dans le territoire profond et superficiel de l'artère sylvienne. (C): angio-TDM abdominale montrant une absence de visualisation du tronc porte et de ses branches de bifurcation remplacées par un cavernome (flèche) avec une splénomégalie

